# Biopsy of breast cancer metastases: patient characteristics and survival

**DOI:** 10.1186/s12885-016-3014-6

**Published:** 2017-01-04

**Authors:** Shlomit Strulov Shachar, Tanya Mashiach, Georgeta Fried, Karen Drumea, Noa Shafran, Hyman B. Muss, Gil Bar-Sela

**Affiliations:** 1 Division of Oncology, Rambam Health Care Campus, Haifa, Israel; 2 Statistical Department, Rambam Health Care Campus, Haifa, Israel; 3 University of North Carolina, Chapel Hill, NC USA; 4 Rappaport Faculty of Medicine, Technion-Israel Institute of Technology, Haifa, Israel; 5 Integrated Oncology and Palliative Care Unit, Rambam Health Care Campus, POB 9602, Haifa, 31096 Israel

**Keywords:** Biopsy, Breast carcinoma, Pathology, Recurrent-metastatic disease, Survival

## Abstract

**Background:**

Discordance in hormone receptors (HR) and human epidermal growth factor receptor 2 (HER2) status between primary tumors and metastatic sites for breast cancer is well established. However, it is uncertain which patient-related factors lead to biopsy when metastases are suspected and whether having a biopsy impacts survival.

**Methods:**

The medical charts of metastatic breast cancer (MBC) patients diagnosed January 2000-August 2014 were retrospectively reviewed. A biopsy was defined as a procedure where tissue was obtained and assessed for both HR and HER2. Both bivariate and multivariate analyses were performed to assess patient characteristics related to biopsy and whether having a biopsy was associated with improved survival.

**Results:**

Of 409 patients suspected of having MBC, 165 (40%) had a biopsy, and 34% of these had discordant HR or HER2 status when compared to the initial diagnosis. In multivariate analysis, having a biopsy was associated with: recurrence in years 2010–2014, disease-free interval of > =3 years, stage 0-IIA at presentation, suspected locoregional recurrence, being HR+/HER2-, or missing HR/HER2 at diagnosis. A similar multivariate analysis revealed that having a biopsy was associated with improved survival (HR = 0.67, *p* = 0.002). The association of biopsy and improved survival was noted in specific subgroups: patients with missing HR and HER2 data at initial diagnosis (*p* = 0.001), those without metastases in liver, lung or brain (*p* = 0.001), and being younger than 70 years old at recurrence (*p* < 0.001).

**Conclusions:**

Specific clinical factors were associated with biopsy at the time of suspected recurrence. Having a biopsy was associated with reduced mortality.

## Background

Breast cancer is the most common cancer among women in the United States, and it is estimated that 246,660 new cancer cases will be diagnosed in 2016 [1]. Despite recent progress in diagnostic and therapeutic approaches of early breast cancer, many patients develop metastases, resulting in about 40,000 deaths annually. Systemic treatment in metastatic breast cancer (MBC), as in early stage breast cancer, is chosen on the basis of estrogen receptor (ER) and progesterone receptor (PR) status and overexpression/amplification of the human epidermal growth factor receptor 2 (HER2). In addition, other factors are routinely used for therapy decisions, such as disease-free interval, site(s) of relapse, and number of metastases [2, 3].

Discordance in hormone receptors and HER2 status between the primary tumor and the metastatic lesion is well documented [4]. A recent review demonstrated a large range of receptor discordance with ER, PR and HER2 discordance of 6–40%, 21–41% and 1–43%, respectively [5]. In addition, a meta-analysis of 48 studies, in which 4200, 2739, and 2987 tumors were evaluated for ER, PR and HER2 discordance, respectively, showed a discordance range of 8–23% [6]. The reasons for discordance may relate to changes in tumor biology between the initial tumor and the metastatic lesion biopsied, heterogeneity within the tumor itself, and/or imperfect accuracy and reproducibility of assays, although the relative contribution of each of these factors to the overall discordance rate is unclear. The unequivocal recommendations for biopsy when metastases are suspected are: solitary metastasis, unusual clinical course, and research [7]. The existent discordance between the primary tumor and recurrent metastasis in ER, PR and HER2 status in portions of tumors has been demonstrated in many studies [5, 8].

The Advanced Breast Cancer (ABC) consensus guidelines recommend biopsying a metastatic lesion, if easily accessible, and confirming the diagnosis, particularly when metastasis is diagnosed for the first time [3]. The National Comprehensive Cancer Network (NCCN) Panel recommended doing a biopsy to confirm recurrence, and repeating ER/PR and HER2 at the metastatic site [2].

Biopsy results may also be related to survival. In a recent Swedish trial, it was concluded that patients with tumor loss of ER or PR during progression have worse survival than patients with retained receptor expression [8]. However, in another retrospective trial, no significant survival difference between patients with discordant versus non-discordant receptors in MBC was seen [9]. Lastly, Curtit et al. reported that patients who had undergone biopsy had survival benefit (median OS 79 months vs 32 months, *p* = 0.0001) [10].

However, it is still unclear whether patient characteristics are associated with having a biopsy and whether having a biopsy is associated with improved survival. Since prospective trials to address these questions are not likely to be performed, we conducted a large retrospective trial to help answer these questions.

## Methods

### Study design

After approval of the study protocol by the institutional ethics committee - Helsinki Committee of Rambam Health Care Campus (RHCC) (Certificate no. 0408-13-RMB) - a retrospective analysis was conducted of all medical records of adult patients treated in the Division of Oncology at RHCC in Haifa, Israel, for metastatic breast carcinoma from January 2000 to August 2014. Eligibility for the study included having metastatic/locoregional non-curable disease, previous treatment for early breast cancer with curative intent (including surgery), and an interval of at least 6 months since the end of adjuvant chemotherapy, radiotherapy, and/or surgery (adjuvant hormonal therapy was allowed). Excluded from the study were patients with *de novo* primary metastatic disease and patients who were not treated with curative intent at first diagnosis (without surgery). Patients were de-identified after data were collected from digital and non-digital records. Data extracted from the medical files included demographics and medical history: age, marital status, number of children, stage (American Joint Committee on Cancer, 7^th^ edition), grade, ER, PR and HER2 status (from initial surgery/biopsy specimen), adjuvant treatment regimen, and type of surgery at initial diagnosis. Data obtained on patients with suspected metastases included the sites of metastasis, if and when a biopsy was done, a pathology report from the biopsy including documentation of metastatic adenocarcinoma, ER, PR and HER2status, and date of death or date of last follow-up. We defined a biopsy as a procedure confirming adenocarcinoma, including reassessment of HR and HER2 status. Patients were defined as not having a biopsy if they did not undergo biopsy, had a biopsy which only histologically confirmed metastases, or did not reassess HR and HER2 status. Positive ER or PR expression was defined as 1% of cells or greater staining for ER or PR. HER2 assessment was scored from 0–3+. In case of overexpression scored at 2+, fluorescent in situ hybridization (FISH) was performed. HER2 was considered positive if overexpression was scored at 3+ in immunohistochemistry, or 2+ in immunohistochemistry, if FISH was positive.

### Statistical analysis

Logistic regression was used to calculate the odds ratios (OR) with 95% confidence intervals (95% CI) and *p* values in bivariate analysis to determine associations between tumor characteristics and likelihood of biopsy. Based on previous theory [11], variables with *p* < =0.1 were included in subsequent Multivariable Forward Stepwise Logistic Regression analysis. The Hosmer-Lemeshow goodness-of-fit statistic [11] was calculated, and the area under the receiver operating characteristic (ROC) curve was used as a measure of model discrimination. Bivariate Cox regression was then used to calculate hazard ratios (HR) with 95% confidence intervals (CI) and *p* values for overall survival (OS). Stratified analysis was used to assess confounding. Variables with *p* < =0.1 were used in multivariate Cox Regression analysis to assess the effect of biopsy on overall survival (OS), as well as the effect of patient and tumor characteristics. Two-tailed *p* values of 0.05 or less were considered statistically significant. Statistical analyses was performed using SPSS (Statistics Products Solutions Services) 21.0 software for Windows.

## Results

### Patient characteristics

The medical records of 409 patients diagnosed with MBC between 2000 and 2014 were evaluated. Of these 409 patients, 165 (40%) patients had a biopsy as defined in this study, while 90 (22%) had only histologic confirmation of metastases.

Median follow-up for metastatic disease was 30.2 months (range, 1.1–154.2 months) for all patients and 42.3 months (range, 1.3–155.8 months) for patients who were alive at the time of chart review. Patient characteristics are shown in Table 1. Median age at metastatic diagnosis was 57.4 years (range, 26.6–88.7 years). Of the 409 patients, 42% were initially diagnosed with stage 0-IIA tumors and 57% with stage IIB-III tumors. Also at initial diagnosis, 186 (45%) patients had grade 1–2 tumors, 146 (36%) had grade 3 tumors (19% data missing), 282 (69%) were HR positive and 92 (22%) were HER2 positive.

**Table 1:** Patient characteristics, bivariate analysis for biopsy, and overall mortality

Variable		No. Patients	No Biopsy	Biopsy			All Cause Mortality		
			No. (%)	No. (%)	*P* value	OR (95% CI)	% total	*P* value	HR (95% CI)
Factors in initial diagnosis									
Year of initial diagnosis	Total	409	244 (60)	165 (40)			80.9		
	1980–99	134	76 (57)	58 (43)	Ref	1.00	85.1	Ref.	1.00
	2000–09	256	154 (60)	102 (40)	0.512	0.87 (0.57–1.33)	79.7	0.000	1.54 (1.22–1.94)
	2010–14	19	14 (74)	5 (26)	0.167	0.47 (0.16–1.37)	68.4	0.000	5.35 (2.95–9.70)
Stage at initial diagnosis	Stage 0-IIA	172	82 (48)	90 (52)	Ref	1.00	73.8	Ref.	1.00
	Stage IIB-III	232	161 (69)	71 (31)	0.000	0.40 (0.27–0.61)	87.1	0.001	1.46 (1.17–1.82)
	Missing	5	1 (20)	4 (80)			40.0		
Grade at initial diagnosis	1.0	16	5 (31)	11 (69)	Ref	1.00	62.5	Ref.	1.00
	2.0	169	103 (61)	66 (39)	0.028	0.29 (0.10–0.88)	80.5	0.167	1.57 (0.83–3.00)
	3.0	146	90 (62)	56 (38)	0.026	0.28 (0.09–0.86)	85.6	0.009	2.37 (1.24–4.52)
	Missing	78	46 (59)	32 (41)			76.9		
HR and HER2 at initial diagnosis	HR+/HER2+	48	38 (79)	10 (21)	Ref.	1.00	75.0	Ref.	1.00
	HR-/HER2-	49	35 (71)	14 (29)	0.379	1.52 (0.60–3.86)	87.8	0.004	1.92 (1.24–3.00)
	HR-/HER2+	44	35 (80)	9 (20)	0.964	0.98 (0.36–2.69)	86.4	0.032	1.65 (1.04–2.61)
	HR+/HER2-	173	99 (57)	74 (43)	0.007	2.84 (1.33–6.07)	80.3	0.090	1.38 (0.95–1.99)
	Missing HR or HER2	95	37 (39)	58 (61)	0.000	5.96 (2.65–13.38)	78.9	0.429	1.17 (0.79–1.75)
Factors in metastatic recurrence									
Time to recurrence	<3 years	145	120 (83)	25 (17)	Ref	1.00	93.1	Ref	1.00
	> = 3 years	264	124 (47)	140 (53)	0.000	5.42 (3.31–8.88)	74.2	0.000	0.58 (0.47–0.73)
Age at recurrence	<70 years	348	207 (59)	141 (41)	Ref	1.00	80.7	Ref	1.00
	> = 70 years	61	37 (60)	24 (40)	0.863	0.95 (0.55–1.66)	82.0	0.004	1.57 (1.16–2.12)
Years of metastatic recurrence	2000–04	116	93 (80)	23 (20)	Ref	1.00	96.6	Ref.	1.00
	2005–09	165	103 (62)	62 (38)	0.002	2.43 (1.40–4.24)	90.3	0.433	1.10 (0.86–1.41)
	2010–14	128	48 (37)	80 (63)	0.000	6.74 (3.77–12.04)	54.7	0.596	1.09 (0.80–1.48)
No of metastatic sites at recurrence	Single site	123	83 (64)	40 (36)	Ref	1.00	78.9	Ref	1.00
	2–3 sites	136	76 (56)	60 (44)	0.056	1.64 (0.99–2.72)	80.1	0.011	1.43 (1.09–1.89)
	>3 sites	150	85 (57)	65 (43)	0.068	1.59 (0.97–2.61)	83.3	0.001	1.57 (1.20–2.06)
Brain metastases	No	376	219 (58)	157 (42)	Ref	1.00	79.8	Ref.	1.00
	Yes	33	25 (76)	8 (24)	0.054	0.45 (0.20–1.02)	93.9	0.000	2.60 (1.79–3.79)
Liver metastases	No	289	176 (61)	113 (39)	Ref	1.00	78.5	Ref.	1.00
	Yes	120	68 (57)	52 (43)	0.427	1.19 (0.77–1.83)	86.7	0.007	1.38 (1.09–1.74)
Lung metastases	No	253	150 (59)	103 (41)	Ref	1.00	79.4	Ref.	1.00
	Yes	156	90 (60)	62 (40)	0.846	0.96 (0.64–1.44)	83.3	0.015	1.32 (1.05–1.64)
HR and HER2 at recurrence	HR+/HER2+	26					69.2	Ref	1.00
	HR-/HER2-	34					85.3	0.008	2.23 (1.24–4.02)
	HR-/HER2+	15					53.3	0.295	1.56 (0.68–3.61)
	HR+/HER2-	90					60.0	0.665	1.13 (0.66–1.92)
	Missing	244					91.0	0.004	2.01 (1.24–3.26)
Site of metastasis at recurrence	No brain, liver or lung	158	89 (56)	69 (44)	Ref	1.00	75.3	Ref.	1.00
	Liver or lung	183	113 (62)	70 (38)	0.310	0.80 (0.52–1.23)	81.4	0.020	1.33 (1.05–1.70)
	Liver & lung	35	17 (49)	18 (51)	0.405	1.37 (0.66–2.84)	91.4	0.000	2.25 (1.52–3.33)
	Brain	33	25 (76)	8 (24)	0.043	0.41 (0.18–0.97)	93.9	0.000	3.22 (2.15–4.81)
LAD at recurrence	No LAD	239	169 (71)	70 (29)	Ref	1.00	83.7	Ref.	1.00
	LAD	170	75 (44)	95 (56)	0.000	3.06 (2.03–4.61)	77.1	0.933	0.99 (0.79–1.24)
Biopsy Status	No	244					91.0	Ref	1.00
	Yes	165					66.1	0.000	0.64 (0.51–0.81)

*HR* hormone receptors, *HER2* human epidermal growth factor receptor 2, *LAD* locally advanced disease, *N/A* not applicable

All patients underwent surgery with curative intent. More than 75% of the patients received chemotherapy (72% anthracycline-based), either as adjuvant or neoadjuvant treatment. Adjuvant trastuzumab was given to only 8% of patients. Adjuvant hormonal therapy was given to 62% of patients. Biopsy sites were: locoregional recurrence (skin, chest wall, breast or lymph nodes (LN)) 77 (47%), bone 33 (20%), liver 26 (16%), lung 14 (8%), abdomen 5, other sites 10.

### Clinical characteristics associated with biopsy

Clinical characteristics found to be significantly associated with having a biopsy are shown in Table 1. Overall, the percentage of patients who underwent biopsy significantly increased between 2000 and 2014. Factors related to having a biopsy included: not having HR or HER 2 status at initial diagnosis (OR = 5.96, *p* < 0.0001), disease-free interval longer than > =3 years (OR = 5.42, *p* < 0.0001), and locally advanced disease (in the breast, LN, skin, or chest wall) (OR = 3.06, *p* < 0.0001). Advanced stages IIB-III (OR = 0.40, *p* < 0.0001) and high grade (grade 3 compared to grade 1 (OR = 0.28, *p* = 0.026)) were associated with fewer biopsies. Of note, patient age at time of suspected metastases was not associated with the biopsy decision.

In multivariate analysis (Table 2), variables that remained significantly associated with biopsy were: year of recurrence (OR = 10.67, *p* < 0.0001 for 2010–2014), disease-free interval of > =3 years (OR = 3.20, *p* < 0.001), stage 0-IIA (OR = 1.89, *p* = 0.012), locally advanced disease (OR = 1.90, *p* = 0.011), HR+/HER2- at initial diagnosis (OR = 2.83, *p* = 0.021), and unknown HR or HER2 at initial diagnosis (OR = 6.78, *p* < 0.001).

**Table 2:** Multivariate analysis : Factors predictive of biopsy

Variable		*P* value	Adjusted OR (95.0% CI for OR)
Years of metastatic recurrence	2000–04		Ref.
	2005–09	.000	3.79 (1.94–7.40)
	2010–14	.000	10.67 (5.13–22.22)
Time to recurrence(years)	<3 years		Ref.
	> = 3 years	.000	3.20 (1.78–5.76)
Stage at initial diagnosis	Stage IIB-III		Ref.
	Stage 0- IIA	.012	1.89 (1.15–3.10)
Site of metastasis at recurrence	No locally advanced disease		Ref.
	Locally advanced disease	.011	1.90 (1.16–3.10)
HR &HER2 at initial diagnosis	HR+/HER2+		Ref.
	HR-/HER2-	.349	1.68 (0.57–4.93)
	HR-/HER2+	.813	1.15 (0.35–3.74)
	HR+/HER2-	.021	2.83 (1.17–6.83)
	Missing HR or/and HER2	.000	6.78 (2.49–18.45)

All other variables were not significant

*HR* hormone receptors, *HER2* human epidermal growth factor receptor 2

### Discordance in hormone receptor and HER2 status among primary and metastatic tumors

**Fig. 1:**
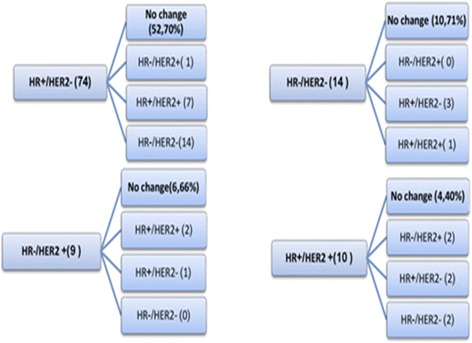
Discordance between initial histology and biopsy (*n* = 107)

In 179 patients whose tumor HR status was evaluated both at primary diagnosis and upon recurrence, HR status changed from positive to negative in 27 (15%) patients, and from negative to positive in 14 (8%) patients. HER2 status was evaluated in 114 patients at both diagnosis and recurrence. HER2 changed from positive to negative in 5 (4%) patients, and from negative to positive in 10 (9%) patients. Figure 1 describes changes from initial pathology to biopsy in 107 patients whose HR and HER2 were measured in both biopsies.

### Factors associated with overall mortality

In bivariate analysis, clinical characteristics associated with overall mortality are summarized in Table 1. At diagnosis, advanced stage (IIB, III), higher grade (grade 3), and having a triple negative or HR-/HER2+ tumor was associated with worse survival. In turn, at the time of metastases, age > =70 years, a disease-free interval of <3 years, multiple metastatic sites, brain, liver and lung metastases, or having triple negative disease was associated with worse survival. Of note, having a biopsy upon recurrence was associated with longer survival (HR = 0.64, *p* < 0.0001).

Multivariate analysis of factors associated with shortened overall survival at initial diagnosis (Table 3) included advanced stage (stage IIB-III) (HR = 1.40, *p* = 0.004), triple negative phenotype (HR = 2.14, *p* = 0.001), and HR+/HER2- phenotype (HR = 1.61, *p* = 0.013). At the time of metastases, age ≥70 years (HR = 1.65, *p* = 0.002), and brain or liver and lung metastasis (HR = 2.85, HR = 2.46, respectively, *p* < 0.001) were associated with shortened overall survival. As in bivariate analysis, not having a biopsy was associated with shorter survival as compared to having a biopsy (HR = 0.67, *p* = 0.002).

**Table 3:** Multivariate analysis: Factors predictive of All Cause mortality

Variable		*P* value	HR Adjusted(95.0% CI for HR)
HR & HER2 at initial diagnosis	HR+/HER2+		Ref.
	HR-/HER2-	.002	2.14 (1.36–3.39)
	HR-/HER2+	.179	1.36 (0.86–2.27)
	HR+/HER2-	.013	1.61 (1.11–2.29
	HR and/or HER2 missing	.065	1.49 (0.98–2.21)
Site of metastasis at recurrence	No brain, liver or lung metastasis		Ref.
	Liver & lung involvement	.000	2.46 (1.68–3.60)
	Brain involvement	.000	2.85 (1.88–4.31)
Age at metastatic recurrence (years)	<70		Ref.
	> = 70	.002	1.64 (1.19–2.24)
Stage at initial diagnosis	Stage 0-IIA		Ref.
	Stage IIB-III	.004	1.40 (1.12–1.76)
Biopsy	No biopsy		Ref.
	Biopsy	.002	0.67 (0.52–0.86)

All other variables were not significant

*HR* hormone receptors, *HER2* human epidermal growth factor receptor 2

### Association of overall survival and biopsy in selected subgroups

Exploratory analysis of the interaction of biopsy and survival was performed in selected subgroups (Table 4). A significant survival benefit was found in patients with missing data on receptor status (*p* = 0.001) at the initial diagnosis, no metastasis in liver and lung or brain (*p* = 0.001), or younger than age 70 (*p* = 0.0001). There was no significant effect on survival among different tumor phenotypes; however, this might be due to the small number of patients in each group.

**Table 4:** Effect of biopsy on survival for selected clinical and pathological variables

		No. Pts.	All Cause Mortality			
			No.	% total	*P* value	HR (95%CI)
	TOTAL	409	331	80.9		
Biopsy at recurrence						
Biopsy	No Bx	244	222	91.0		1.00
	Bx	165	109	66.1	0.000	0.64 (0.51–0.81)
HR & HER2at initial diagnosis						
HR+/HER2+	No Bx	38	30	78.9		1.00
	Bx	10	6	60.0	0.499	0.74 (0.30–1.79)
HR-/HER2-	No Bx	35	33	94.3		1.00
	Bx	14	10	71.4	0.236	0.65 (0.32–1.33)
HR-/HER2+	No Bx	35	33	94.3		1.00
	Bx	9	5	55.6	0.562	0.75 (0.29–1.98)
HR+/HER2-	No Bx	99	90	90.9		1.00
	Bx	74	49	66.2	0.097	0.74 (0.52–1.06)
HR and/or HER2 missing	No Bx	37	36	97.3		1.00
	Bx	58	39	67.2	0.001	0.45 (0.28–0.71)
Site of metastasis at recurrence						
No liver & lung or brain metastases	No Bx	202	182	90.1		1.00
	Bx	139	86	61.9	0.001	0.63 (0.49–0.82)
Liver & lung metastases	No Bx	17	15	88.2		1.00
	Bx	18	17	94.4	0.539	0.80 (0.39–1.63)
Brain metastases	No Bx	25	25	100.0		1.00
	Bx	8	6	75.0	0.196	0.55 (0.22–1.36)
Age at metastatic recurrence						
Age <70 years	No Bx	207	189	91.3		1.00
	Bx	141	92	65.2	0.000	0.62 (0.48–0.80)
Age >=70 years	No Bx	37	33	89.2		1.00
	Bx	24	17	70.8	0.587	0.85 (0.47–1.54)
Stage at initial diagnosis						
Stage 0-IIA	No Bx	82	71	86.6		1.00
	Bx	90	56	62.2	0.025	0.67 (0.47–0.95)
Stage IIB-III	No Bx	161	151	93.8		1.00
	Bx	71	51	71.8	0.034	0.71 (0.51–0.97)

*HR* hormone receptors, *HER2* human epidermal growth factor receptor 2, *Bx* biopsy

## Discussion

The primary purposes in doing a biopsy when MBC is suspected are to confirm the diagnosis of metastasis and to reassess HR and HER2 status, two key factors that influence subsequent treatment. In a prospective trial that included 64 women with a history of prior breast cancer and suspected new metastases, a non-breast cancer diagnosis was found in 14, and 10% had benign findings [12]. Of the 205 patients prospectively included in the BRITS trial, 9% did not have recurrent disease on biopsy [13]. In our study, 33% of patients had a biopsy only for histological confirmation of MBC. We also found that, in recent years, physicians ordered significantly more biopsies (OR = 10.67, *p* < 0.0001 for 2010–2014), perhaps due to the fact that recent studies have emphasized that receptor status can change [10, 14].

The main purpose of a biopsy is to reassess the HR and HER2 status of the metastatic disease. In our study, a biopsy that included confirmation of HR and HER2 status was associated with a significant survival benefit (*p* = 0.001). In a study by Amir et al. [14], there were discordant findings between findings at initial diagnosis and findings after metastasis in 37.6% of patients, resulting in a treatment change in 14% of patients. However, no change in OS was noted between concordant and discordant groups in that study [14]. Different results were seen in a study in China, which showed worse OS for patients who had receptors discordance in the biopsy [15]. However, in a review of 13 recent major studies regarding biopsy in MBC, the impact of this procedure on survival was not clear [16].

Improved survival for patients whose metastases retained ER or PR expression as compared to those with loss of ER or PR expression was shown by Karlsson et al. in a cohort of 177 patients with MBC [8], and in a pooled analysis of two prospective studies (BRITS and DESTINY studies) that included 289 MBC patients, where a gain of HR or HER2 expression led to most changes in treatment approaches [17]. We demonstrated that a biopsy that included confirmation of HR and HER2 was associated with lower mortality. However, the major benefit was seen mainly in patients who had missing data on HR or HER2 status at initial diagnosis (*p* = 0.001).

We offer some explanations for the association of undergoing biopsy and improved survival: biopsy may show discordance with initial findings that may be associated with improved treatment selection, in turn, resulting in a higher response rate, longer progression free interval, and possibly improved survival. This explanation is supported by recent data showing that molecular subtyping and gene modules of post-relapse biopsy are associated with survival [18]. In our study, 8% of patients who were initially HR- became HR+, suggesting that endocrine therapy should be considered. At the same time, 15% of patients who were HR+ at diagnosis became HR- at biopsy and would likely not have benefitted from endocrine therapy; for these patients, chemotherapy would be a better choice. In 9% of patients, HER2- changed to HER2+, a change that can modify the treatment plan to include anti-HER2 directed therapy; anti-HER2 directed therapies significantly improved survival for patients with HER2+ MBC [19, 20]. Of note, a small group, 4% of patients, changed from HER2+ to HER2- and most likely would not have benefitted from anti-HER2 therapy. Another possible explanation for what we observed in our sample is that physicians choose to biopsy patients whose clinical scenarios are associated with improved survival, such as stage at initial presentation and longer disease-free intervals (Table 2). To test this scenario, a prospective randomized control trial would be needed.

Although no difference was found in biopsy percentages by age groups, patients <70 years who had a biopsy were found to have improved survival in subgroup analysis (*p* < 0.0001). This finding might be partially due to competing causes of mortality in older patients and the fact that older patients are more likely to have HR+ tumors and HR+ metastases [21]. In a large study with a long follow-up period (28 years), 30% of metastatic breast cancer patients >70 years died from non-breast cancer causes [22].

Our study has several limitations. First, it is retrospective and we assume but cannot be certain that experienced clinicians chose to biopsy patients with suspected metastases with a more favorable prognosis. Second, we do not have data on how treatment decisions were affected in patients with discordant biopsy findings. Third, our study was not sufficiently powered to check discordance of HR and HER2 for different metastatic sites. In one study that examined discordance in 233 distant breast cancer metastases at different sites (76 skin, 63 liver, 43 lung, 44 brain, 7 gastro-intestinal), receptor conversion seemed to occur mostly in liver and brain metastases; however, this was significant only for PR conversion [23].

## Conclusions

Our data confirm the high discordance rate between findings at initial breast cancer diagnosis and findings at metastases. Our findings suggest that patients most likely to benefit from biopsy are those without liver, lung or brain metastases, are younger than 70 years of age, and with missing receptors status at initial diagnosis. Recent American Society of Clinical Oncology (ASCO) guidelines [24] suggest that, in patients with accessible metastases, biopsy for confirmation of metastases and retesting of estrogen receptor, progesterone receptor, and HER2 status should be offered. However, evidence is lacking to determine whether modifying anticancer therapy on the basis of a change in receptor status influences clinical outcomes [24]. The ASCO panel consensus was to preferentially use the ER, PR, and HER2 status of the metastasis to direct therapy, if supported by the clinical scenario and the patient’s goals. In addition, tissues from metastases may be used to assess molecular differences not limited to receptor level but also to investigate DNA, RNA, protein and functional pathway levels and to look for actionable mutations that might allow for novel new therapies [5, 25]. Our findings support current recommendations and show that, in selected patients, a biopsy at metastatic recurrence that included HR and HER2 status was associated with significantly reduced mortality.

## Abbreviations

CI: Confidence intervals
ER: Estrogen receptor
FISH: Fluorescent in situ hybridization
HER2: Human epidermal growth factor receptor 2
HR: Hazard ratio
HR: Hormone receptor
MBC: Metastatic breast cancer
OR: Odds ratio
OS: Overall survival
PR: Progesterone receptor
RHCC: Rambam Health Care Campus
